# Screen Time, Sociodemographic Factors, and Psychological Well-Being Among Young Children

**DOI:** 10.1001/jamanetworkopen.2023.54488

**Published:** 2024-03-05

**Authors:** Soyang Kwon, Bridget Armstrong, Nina Wetoska, Selin Capan

**Affiliations:** 1 Department of Pediatrics, Feinberg School of Medicine, Northwestern University, Chicago, Illinois; 2 Department of Exercise Science, Arnold School of Public Health, University of South Carolina, Columbia, South Carolina; 3 Buehler Center for Health Policy and Economics, Feinberg School of Medicine, Northwestern University, Chicago, Illinois

## Abstract

**Importance:**

Young children’s screen time increased during the COVID-19 lockdown in 2020, but it is unknown whether their screen time returned to prepandemic levels in 2021. Knowledge of the relationship between screen time and child development and health will inform prevention and intervention targets and strategies.

**Objective:**

To evaluate screen time by family income and race and ethnicity in the prepandemic (ie, 2018, 2019) and pandemic (ie, 2020, 2021) periods and to examine the relationship between screen time and psychological well-being among young children in the US.

**Design, Setting, and Participants:**

Cross-sectional population-based web or mail survey study of 2018-2021 National Survey of Children’s Health participants aged 6 months to 5 years.

**Exposure:**

Daily screen time (<1, 1, 2, 3, or ≥4 h/d) reported by participants’ primary caregivers.

**Main outcomes and Measures:**

Flourishing and externalizing behaviors, as indicators of psychological well-being. All analyses were weighted, accounting for the complex survey sample design.

**Results:**

Of the 48 775 participants (50.7% female), the proportion of those with high screen time (defined as ≥1 h/d for children aged 6 months-1 year and ≥2 h/d for children aged 2-5 years) was 48.5% (95% CI, 46.3%-50.7%) in 2018, 49.2% (95% CI, 47.0%-51.5%) in 2019, 55.3% (95% CI, 53.4%-57.2%) in 2020, and 50.0% (95% CI, 48.3%-51.6%) in 2021. Among children living in poverty (federal poverty level <100%), the proportion of those with high screen time was 48.7% (95% CI, 42.8%-54.6%) in 2018, 52.0% (95% CI, 45.4%-58.6%) in 2019, 60.9% (95% CI, 55.4%-66.4%) in 2020, and 58.9% (53.7%-64.1%) in 2021. Adjusted odds ratio of flourishing was 0.66 (95% CI, 0.51-0.85), 0.81 (95% CI, 0.66-0.99), 0.68 (95% CI, 0.52-0.88), and 0.53 (95% CI, 0.42-0.69) for less than 1, 2, 3, and 4 or more hours per day vs 1 hour per day of screen time, respectively, among children aged 3 to 5 years. No association between screen time and flourishing was found among children aged 6 months to 2 years. An adjusted externalizing behavior score was higher by 0.2 points (95% CI, −0.1 to 0.5), 0.5 points (95% CI, 0.3 to 0.8), 1.3 points (95% CI, 1.0 to 1.6), and 2.1 points (95% CI, 1.7 to 2.5) for less than 1, 2, 3, and 4 or more hours per day vs 1 hour per day of screen time, respectively, among children aged 3 to 5 years.

**Conclusions and Relevance:**

In this multiyear cross-sectional study of a representative sample of young children in the US, the increased prevalence of high screen time in 2020 returned to prepandemic levels in 2021; however, it remained elevated in children living in poverty. Two hours or more of daily screen time was associated with lower psychological well-being among preschool-aged children.

## Key Points

**Question:**

Is there a relationship between screen time and psychological well-being among young children?

**Finding:**

In a nationally representative sample of 48 775 young US children, children who had 2 or more hours a day of screen time had lower levels of psychological well-being indicators, compared to children who had 1 hour a day of screen time.

**Meaning:**

Study findings suggest an association between 2 hours or more of daily screen time and the psychological well-being of young children.

## Teaser

This cross-sectional study uses data from the 2018 to 2021 National Survey of Children’s Health of children aged 6 months to 5 years to evaluate sociodemographic factors of daily screen time in the COVID-19 prepandemic (2018 and 2019) and pandemic (2020 and 2021) periods and examines the association between screen time and psychological well-being in this population.

## Introduction

Screen time refers to the amount of time that individuals spend watching television, playing video games, and using computers, mobile phones, tablets, or other electronic devices.^1^ Screen time among young children (age 5 years and under) has been increasing globally.^2^ This trend escalated^3,4^ during the first year of the COVID-19 pandemic emergency period (March 2020-May 2023). Hedderson et al^4^ reported that increased screen time in the earlier pandemic period (December 2020-April 2021) did not return to that study’s prepandemic levels (July 2019-March 2020) in the later pandemic period (May-August 2021) among US children aged 4 to 12 years. Nonetheless, it is largely unknown whether increased screen time levels among young children continued into 2021 and beyond. The pandemic also amplified health disparities by income and race and ethnicity in the US.^5,6^ However, few studies have investigated whether the pandemic disproportionally affected screen use behaviors of young children from low-income and minoritized racial and ethnic backgrounds. This knowledge is crucial to plan interventions for populations at risk of high screen time and address health disparities.

Several studies^7,8,9,10,11,12,13,14,15,16^ have suggested potential negative impacts of high screen time on children’s development. Screen use was reported to be inversely associated with psychological well-being among US children and adolescents.^17^ Specifically, flourishing and behavioral difficulties are key indicators of young children’s psychological well-being.^18^ Flourishing is not just the absence of problems, but the presence of protective factors, such as positive emotions, positive relationships, and cognitive and adaptive functioning.^18,19,20,21^ Flourishing was reported to reduce the risk of antisocial behaviors and mortality later in life.^18,22,23^ One study^17^ found that 4 hours per day or longer of screen time was associated with lack of curiosity, 1 of the flourishing dimensions, among US children aged 2 to 5 years. Externalizing behaviors are behavioral problems that are manifested in children’s outward behavior, such as hyperactive and aggressive behaviors, and conceptualized to reflect maladjustment to the external environment.^24,25^ Externalizing behaviors concurrently negatively affect learning and relationships,^26^ with potential long-term risks for juvenile delinquency and adult violence.^25^ A meta-analysis^27^ found a small but significant correlation (*r* = 0.11) between screen time and externalizing behaviors in children 12 years or younger. However, population-based evidence specifically for young children is lacking, limiting the external validity of the findings.

To address these gaps, we aimed to evaluate screen time across 4 years, 2018 to 2021, in a nationally representative sample of young US children (aim 1) and examine cross-sectional dose-response relationships between screen time and psychological well-being (aim 2). We hypothesized that changes in screen time of young US children during the prepandemic and pandemic periods would differ by family income and racial and ethnic background and that young children who have higher screen time would have lower psychological well-being.

## Methods

### Study Participants

In this cross-sectional study, secondary data analysis was conducted using the 2018, 2019 (prepandemic period) and 2020, 2021 (pandemic period) National Survey of Children’s Health (NSCH) data. The NSCH reports sample size, margin of sampling error, weighting attributes, the full text of the questions, answer options, the survey mode, and the population under study in accordance with the American Association for Public Opinion Research reporting guidance. Funded and directed by US Maternal and Child Health Bureau of the Health Resources and Services Administration, the NSCH was a web- and mail-based national survey of noninstitutionalized US children aged 6 months to 17 years, collecting information on health and well-being. The NSCH was fielded by the US Census Bureau annually from June or July to January. The NSCH randomly sampled addresses from an extract of the US Census Bureau’s master address file including 50 states and the District of Columbia to mail a screener questionnaire. Based on screener questionnaire responses, 1 child from each household was randomly selected and invited to complete a survey questionnaire. An adult who was familiar with the participating child’s health and health care (primary caregiver) completed the questionnaire online or via mail in English or Spanish. Annual Response rates ranged from 37% to 42% across the 4 years.

The 2018-2021 NSCH data sets contained data from 49 610 children 5 years or younger (Figure). For aim 1, we excluded children (1.7%) with missing screen time responses. For aim 2, we further excluded children with health conditions (ie, blindness, cerebral palsy, deafness, Down syndrome, epilepsy, or intellectual disability) that might affect screen time and/or psychological well-being^17^ or with missing responses to those health condition questions. We also excluded children (<1.0%) with missing responses to psychological well-being variables. We did not exclude children diagnosed with autism spectrum disorder (ASD) and/or developmental delay. Rather, we conducted separate analyses for those subgroups. The institutional review board of the Ann & Robert H. Lurie Children’s Hospital of Chicago determined the study to be exempt from review and waived the requirement to obtain informed consent because it used existing deidentified, publicly available data. This study followed the Strengthening the Reporting of Observational Studies in Epidemiology (STROBE) reporting guideline.

**Figure.**
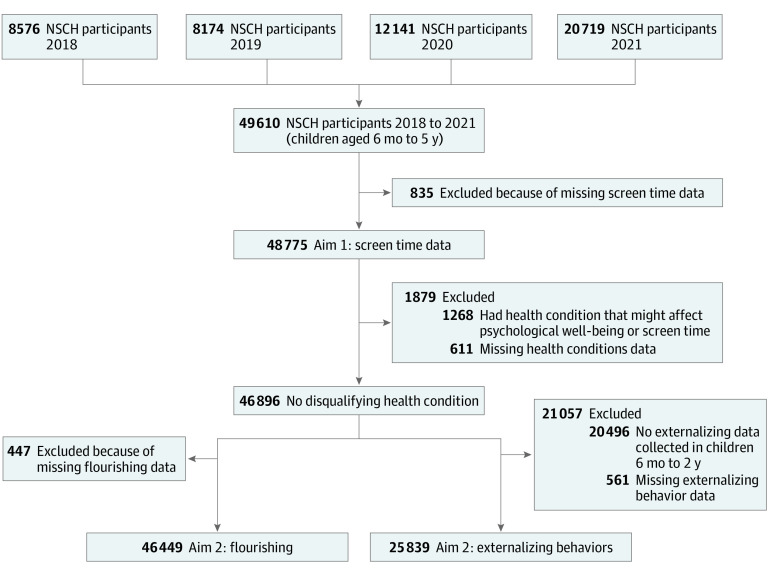
Flowchart of Exclusion Criteria for the 2018 to 2021 National Survey of Children’s Health Participants Aged 6 Months to 5 Years Health conditions refers to blindness, cerebral palsy, deafness, Down syndrome, epilepsy, and intellectual disability.

### Outcome: Psychological Well-Being

Psychological well-being indicators included flourishing and externalizing behaviors. Consistent with previous studies,^19,21^ flourishing was assessed using 4 NSCH items: (1) “How often is this child affectionate and tender with you?” (affection), (2) “How often does this child bounce back quickly when things do not go their way?” (resilience), (3) “How often does this child show interest and curiosity in learning new things?” (curiosity), and (4) does this child smile and laugh?” (affect). Each item was rated on a 4-point scale: “never,” “sometimes,” “usually,” and “always.” Those with “always” or “usually” responses to all 4 questions were categorized as flourishing.^19,21^

Externalizing behaviors were assessed among children 3 years of age or older, using 4 hyperactivity questions and 2 aggression questions.^28^ Hyperactivity questions were (1) “When excited or all wound up, how often can this child calm down quickly?” (reverse-scored); (2) “Compared to other children his or her age, how often is this child able to sit still?” (reverse-scored); (3) “How often is this child easily distracted?”; and (4) “How often does this child keep working at something until he or she finished?” (reverse-scored). Aggression questions were (1) “How often does this child lose control of his or her temper when things do not go his or her way?” and (2) “How often does this child become angry or anxious when going from one activity to another?” Each item was rated on a 5-point scale: “never,” “sometimes,” “about half the time,” “most of the time,” and “always” (scored 1-5, respectively). The hyperactivity and aggression scores were calculated by summing the item scores. Externalizing behavior scores were calculated by summing hyperactivity and aggression scores (range, 6 to 30).

### Exposure: Daily Screen Time

Consistent with a prior study,^29^ we used a single NSCH question to assess screen time: “On most weekdays, about how much time did this child spend in front of a television, computer, cellphone, or other electronic devices watching programs, playing games, accessing the internet or using social media?” Response options included “less than 1 hour,” “1 hour,” “2 hours,” “3 hours,” and “4 or more hours.” Less than 1 hour for children aged 6 months to 1 year and less than 1 hour or 1 hour for children aged 2 to 5 years were considered as meeting the American Academy of Pediatrics (AAP) screen time recommendations.^30^ Those not meeting the recommendation were considered to have high screen time.

### Other Variables

To examine screen time by family income, we used the federal poverty level variable calculated based on household member and household income information and categorized it into less than 100% (below poverty level), 100 to less than 200%, 200 to less than 400%, and 400% or more.^31^ To examine screen time by major racial and ethnic groups in the US, we used primary caregiver-reported child race (American Indian or Alaska Native alone, Asian alone, Black or African American alone, Native Hawaiian and other Pacific Islander alone, multirace, White alone) and ethnicity (Hispanic or non-Hispanic) information. Those who responded as Hispanic to the ethnicity question were categorized as Hispanic. Among those who responded as non-Hispanic, race and ethnicity was categorized into non-Hispanic Asian, non-Hispanic Black, non-Hispanic multirace, non-Hispanic White, and other (American Indian or Alaska Native, Native Hawaiian and other Pacific Islander).

In addition, we considered the following multilevel sociodemographic factors based on the literature^19,32,33^: child’s age and sex, primary caregiver’s education attainment (≤high school graduation, technical school or some college, or ≥4-year college degree), marital status (married, not married but living with a partner, never married, or divorced, separated, or widowed), mental or emotional health (excellent to good or fair to poor), and emotional support for parenting (yes or no response to the question, “During the past 12 months, was there someone that you could turn to for day-to-day emotional support with parenting or raising children?”), primary home language (English or non-English), and neighborhood support (supportive or not supportive). Neighborhood support was defined using the NSCH neighborhood support indicator definition^31^: responses (5-point Likert scale from “definitely agree” to “definitely disagree”) to 3 statements: (1) “People in this neighborhood help each other out”; (2) “We watch out for each other’s children in this neighborhood”; and (3) “When we encounter difficulties, we know where to go for help in our community.” Children were considered living in a supportive neighborhood if their caregiver reported “definitely agree” to at least 1 of the items above and “somewhat agree” or “definitely agree” to the other 2 items.^31^

### Statistical Analysis

All analyses accounted for the complex survey sample design, using SAS, version 9.4 (SAS Institute Inc). Distribution analyses were performed to compare the characteristics of participants with and without missing screen time data. For the home language variable with 0.4% missing data, we excluded the missing observations from analysis. For other sociodemographic variables with >0.5% missing data, we created a missing category. The prevalence of high screen time was calculated by family income categories, racial and ethnic groups, and other participant characteristics. χ^2^ Tests and *t* tests were performed to compare the psychological well-being variables by screen time and sociodemographic variables. A 2-sided *P* = .05 was considered statistically significant.

All analyses were weighted, accounting for the following complex survey sample design. Sample strata were created by the state of residence. Then the sample frame used administrative records-based flags to identify 4 mutually exclusive strata (strata 1A, 1B, 2A, and 2B). Addresses that were explicitly linked to children using administrative records were assigned to stratum 1. Within stratum 1, if a linked child was 5 years old or younger, the address was assigned to stratum 1A; otherwise, the address was assigned to stratum 1B. Addresses that were probabilistically linked to children using administrative records and block group characteristics were assigned to stratum 1A. The remaining addresses were assigned to stratum 2B. These 4 strata were combined into 2: 1 (1A and 1B) and 2 (2A and 2B) Therefore, 2 stratum identifiers were accounted for to estimate variance: state of residence and households flagged with children. Child-level weights calculated for final weight for interviewed children were also accounted for.

As age-specific multivariable logistic regression models indicated a significant association between screen time and flourishing in 3-, 4-, and 5-year-olds but not in 6-month-olds or 1- or 2-year-olds, we built separate logistic regression models for age groups (6 months-2 years and 3-5 years) including the independent variable of screen time (<1 h, 1 h, 2 h, 3 h, or ≥4 h/d), adjusted for the sociodemographic variables. Multivariable linear regression analysis was conducted for the externalizing behavior score. In examining a dose-response relationship, we selected 1 hour per day of screen exposure as a reference group because it showed the highest probability of flourishing and the lowest externalizing behavior score. Subgroup analyses were conducted for children with ASD, children with developmental delay (but without ASD), and by sex.

## Results

A total of 48 775 participants (49.3% male and 50.7% female) were included in analysis (Table 1). Compared to the 48 775 included participants, the 835 excluded participants were more likely to be Hispanic or non-Hispanic Black and had lower income (eTable 1 in Supplement 1).

**Table 1.** Prevalence of High Screen Time by Characteristics of Participants Aged 6 Months to 5 Years^a^^,^^b^

	Sample, No.	Proportion of children with high screen time, % (95% CI)^a^	χ^2^ *P* value
All	48 775	50.7 (49.7-51.7)	
Sex			
Male	25 320	51.2 (49.8-52.6)	.35
Female	23 455	50.2 (48.8-51.7)	
Race and ethnicity^c^			
Hispanic	6336	55.8 (53.0-58.6)	<.001
Non-Hispanic Asian	2523	51.6 (47.5-55.8)	
Non-Hispanic Black	2780	64.4 (61.3-67.6)	
Non-Hispanic multirace	3833	50.5 (47.2-53.8)	
Non-Hispanic White	32 858	45.0 (44.0-46.1)	
Other	445	55.8 (53.0-58.6)	
Federal poverty level^d^			
<100%	4859	55.1 (52.1-58.1)	<.001
100% to <200%	8162	55.8 (53.4-58.2)	
200% to <400%	17 576	52.3 (50.6-54.0)	
≥400%	18 178	42.6 (41.1-44.1)	
Caregiver’s education			
≤High school	6561	53.7 (51.1-56.3)	<.001
Technical school or some colleague	12 892	58.7 (56.8-60.5)	
≥4-y College degree	29 322	44.9 (43.7-46.1)	
Caregiver’s marital status			
Married	39 012	48.3 (47.2-49.5)	<.001
Not married, but living with a partner	3404	53.5 (49.5-57.5)	
Never married	2554	60.3 (56.5-64.1)	
Divorced, separated, or widowed	2783	62.2 (58.4-65.9)	
Missing	1022	52.6 (45.6-59.7)	
Caregiver’s mental or emotional health			
Excellent to good	45 054	50.1 (49.1-51.2)	.001
Poor to fair	2736	59.6 (55.1-64.2)	
Missing	985	52.2 (45.0-59.5)	
Caregiver having emotional support for parenting			
Yes	41 825	49.7 (48.7-50.8)	<.001
No	6648	55.3 (52.5-58.2)	
Missing	302	41.4 (30.2-52.5)	
Primary household language^e^			
English	44 726	50.8 (49.8-51.9)	.89
Non-English	3854	50.6 (47.2-54.0)	
Supportive neighborhood^f^			
Yes	28 008	46.1 (44.8-47.4)	<.001
No	19 766	56.4 (54.9-58.0)	
Missing	1001	46.4 (38.8-54.0)	

^a^ Data from the 2018-2021 National Survey of Children’s Health.

^b^ High screen time was defined as screen time less than 1 hour per day for children aged 6 months to 1 year and screen time 2 or more hours per day for children aged 2 to 5 years.

^c^ Race and ethnicity were ascertained by primary caregiver-reported child race (American Indian or Alaska Native alone, Asian alone, Black or African American alone, Native Hawaiian and Other Pacific Islander alone, White alone, and multirace) and ethnicity (Hispanic or non-Hispanic) information. Those who responded as Hispanic to the ethnicity question were categorized as Hispanic. Among those who responded as non-Hispanic, race and ethnicity were categorized into non-Hispanic Asian, non-Hispanic Black, non-Hispanic multirace, non-Hispanic White, and other (American Indian or Alaska Native or Native Hawaiian and Other Pacific Islander).

^d^ Calculated based on household member and household income information; <100% indicates poverty.

^e^ Missing data (n = 195) were excluded.

^f^ Neighborhood support was evaluated using responses (5-point Likert scale from “definitely agree” to “definitely disagree”) to 3 statements: (1) “People in this neighborhood help each other out”; (2) “We watch out for each other’s children in this neighborhood”; and (3) “When we encounter difficulties, we know where to go for help in our community.” Children were considered living in a supportive neighborhood if their caregiver reported “definitely agree” to at least 1 of the items above and “somewhat agree” or “definitely agree” to the other 2 items.

Overall, 50.7% (95% CI, 49.7%-51.7%) of the sample had high screen time (Table 1). Compared to 2018 and 2019, children aged 2 to 5 years, children living in federal poverty level 200% or more, and Hispanic and non-Hispanic White children had a significantly higher prevalence of high screen time in 2020, but not in 2021; however, children living in federal poverty level below 100% had a significantly higher prevalence rate in both 2020 and 2021 and non-Hispanic Black children had a significantly higher prevalence rate in 2021 (Table 2).

**Table 2.** The Prevalence of High Screen Time Over Survey Years Among Participants Aged 6 Months to 5 Years^a^^,^^b^

	Sample, No.	Prevalence, % (95% CI)			
		2018	2019	2020	2021
All	48 775	48.5 (46.3-50.7)	49.2 (47.0-51.5)	55.3 (53.4-57.2)	50.0 (48.3-51.6)
Age					
6-11 mo	5054	17.6 (13.4-21.8)	21.8 (15.9-27.7)	18.4 (14.6-22.2)	17.3 (13.9-20.6)
1 y	6320	44.5 (38.9-50.1)	47.8 (41.9-53.6)	47.0 (42.0-52.0)	49.7 (42.3-54.1)
2 y	9783	51.2 (46.4-56.0)	46.4 (41.1-51.6)	56.4 (52.3-60.6)	48.2 (44.7-51.6)
3 y	9146	58.2 (53.1-63.3)	52.1 (46.7-57.5)	64.6 (60.2-68.9)	56.9 (53.2-60.6)
4 y	9232	58.5 (53.3-63.6)	65.2 (60.5-69.8)	71.6 (67.8-75.3)	61.2 (57.2-65.3)
5 y	9240	57.4 (52.1-62.6)	59.7 (54.7-64.7)	68.6 (64.0-73.3)	62.2 (58.4-66.1)
Federal poverty level^c^					
<100%	4859	48.7 (42.8-54.6)	52.0 (45.4-58.6)	60.9 (55.4-66.4)	58.9 (53.7-64.1)
100 to <200%	8162	52.2 (46.9-57.5)	58.1 (52.9-63.3)	56.5 (52.1-60.9)	56.9 (53.1-60.6)
200 to <400%	17 576	51.2 (47.5-54.9)	50.3 (46.6-54.0)	57.0 (53.8-60.2)	50.3 (47.7-53.1)
≥400%	18 178	42.4 (39.1-45.7)	39.3 (36.0-42.6)	49.2 (46.2-52.2)	39.5 (37.0-42.1)
Race and ethnicity^d^					
Hispanic	6336	53.4 (47.1-59.6)	54.8 (48.6-61.0)	61.9 (56.9-67.0)	53.4 (48.9-57.9)
Non-Hispanic Asian	2523	52.0 (43.0-61.1)	55.4 (47.2-63.7)	55.3 (46.9-63.8)	44.2 (37.7-50.6)
Non-Hispanic Black	2780	54.7 (47.4-61.9)	65.1 (58.3-72.0)	65.7 (59.7-71.7)	72.2 (67.5-76.9)
Non-Hispanic multirace	3833	51.2 (44.2-58.2)	44.0 (36.7-51.2)	56.2 (49.9-62.5)	62.5 (50.7-65.2)
Non-Hispanic White	32 858	44.5 (42.2-46.7)	43.1 (40.8-45.3)	49.4 (47.2-51.5)	43.1 (41.4-44.9)
Other^e^	445	NA	NA	NA	NA

Abbreviation: NA, not applicable.

^a^ Data from the 2018-2021 National Survey of Children’s Health.

^b^ High screen time was defined as screen time less than 1 hour per day for children aged 6 months to 1 year and screen time 2 or more hours per day for children aged 2 to 5 years.

^c^ Calculated based on household member and household income information; less than 100% indicates poverty.

^d^ Race and ethnicity were ascertained by primary caregiver-reported child race (American Indian or Alaska Native alone, Asian alone, Black or African American alone, Native Hawaiian and Other Pacific Islander alone, White alone, and multirace) and ethnicity (Hispanic or non-Hispanic) information. Those who responded as Hispanic to the ethnicity question were categorized as Hispanic. Among those who responded as non-Hispanic, race and ethnicity were categorized into non-Hispanic Asian, non-Hispanic Black, non-Hispanic multirace, non-Hispanic White, and other (American Indian or Alaska Native or Native Hawaiian and Other Pacific Islander).

^e^ Due to the small sample size, year-specific analysis is not provided.

A Cronbach α coefficient was 0.66 for the 4 flourishing items and 0.75 for the 6 externalizing behavior items. Children aged 3 to 5 years had significantly higher externalizing behavior scores during the pandemic period, compared to the prepandemic period (eTable 2 in Supplement 1). Children who had high screen time had a significantly lower flourishing percentage and a significantly higher externalizing behavior score, compared to those who did not (Table 3). After adjusting for the covariates, the adjusted odds ratio (AOR) of flourishing was 1.15 (95% CI, 0.83-1.59), 0.76 (95% CI, 0.53-1.09) and 1.08 (95% CI, 0.74-1.58) for less than 1, 2, 3, and 4 or more hours per day of screen time, respectively, vs 1 hour per day among children aged 6 months to 2 years (Table 4; eTable 3 in Supplement 1 for a complete model). The AOR of flourishing was 0.66 (95% CI, 0.51-0.85) for less than 1, 0.81 (95% CI, 0.66-0.99), 0.68 (95% CI, 0.52-0.88), and 0.53 (95% CI, 0.42-0.69) for 2, 3, and 4 or more hours per day of screen time, respectively, vs 1 hour per day among children aged 3 to 5 years. The adjusted externalizing behavior score was higher by 0.2 points (95% CI, −0.1 to 0.5), 0.5 (95% CI, 0.3-0.8) points, 1.3 (95% CI, 1.0-1.6) points, and 2.1 (95% CI, 1.7-2.5) points for less than 1, 2, 3, and 4 or more hours per day of screen time, respectively, vs 1 hour per day among children aged 3- to 5 years. Sex-specific analysis showed similar β coefficients between males (β = 0.5 [95% CI, 0.1-0.8], 1.1 [95% CI, 0.7-1.5], and 2.1 [95% CI, v1.5-2.6] for 2, 3, and ≥4 h/d of screen time, respectively, vs 1 h/d) and females (β = 0.7 [95% CI, 0.4-1.0], 1.5 [95% CI, 1.1-2.0], and 2.1 [95% CI, 1.5-2.7] for 2, 3, and ≥4 h/d of screen time, respectively, vs 1 h/d).

**Table 3.** Flourishing and Externalizing Behavior Score by Screen Time Among Participants Aged 6 Months to 5 Years^a^^,^^b^

	Children without high screen time	Children with high screen time	*P* value
Flourishing for age 6 mo-5 y , % (95% CI) (n = 46 449)^c^	85.5 (84.3-86.6)	81.4 (80.2-82.7)	<.001
Usually or always affectionate and tender (affection)	97.2 (96.7-97.7)	96.8 (96.4-97.3)	.30
Usually or always bounces back quickly when things do not go their way (resilience)	90.0 (89.1-91.0)	86.1 (85.1-87.2)	<.001
Usually or always interested and curious in learning new things (curiosity)	96.6 (95.9-97.2)	95.0 (94.3-95.8)	.002
Usually or always smiles and laughs (affect)	98.2 (97.8-98.6)	99.3 (99.0-99.5)	<.001
Externalizing behavior score for age 3-5 y, mean (95% CI) (n = 25 839)^d^	13.1 (13.0-13.3)	14.2 (14.0-14.3)	<.001
Hyperactivity score	9.0 (8.9-9.1)	9.8 (9.7-9.9)	<.001
Aggression score	4.1 (4.0-4.1)	4.4 (4.3-4.4)	<.001

^a^ Data from the 2018-2021 National Survey of Children’s Health.

^b^ High screen time was defined as screen time less than 1 hour per day for children aged 6 months to 1 year and screen time 2 or more hours per day for children aged 2 to 5 years.

^c^ Scores range from 1 to 4 (never, sometimes, usually, and always, respectively). Those responding always or usually to all 4 questions were categorized as flourishing.

^d^ Scores range from 1 to 5 (never, sometimes, about half the time, most of the time, and always). The hyperactivity (based on 4 questions) and aggression (based on 2 questions) scores were calculated by summing the item scores. Externalizing behavior scores were calculated by summing hyperactivity and aggression scores.

**Table 4.** Associations of Screen Time With Flourishing and Externalizing Behavior Score Among Participants Aged 6 Months to 5 Years^a^

	Screen time, h/d				
	<1	1	2	3	≥4
Odds of flourishing, age 6 mo-2 y, OR (95% CI)^b^	1.26 (0.95 to 1.66)	[Reference]	1.15 (0.83 to 1.59)	0.76 (0.53 to 1.09)	1.08 (0.74 to 1.58)
Odds of flourishing, age 3-5 y, OR (95% CI)	0.66 (0.51 to 0.85)	[Reference]	0.81 (0.66 to 0.99)	0.68 (0.52 to 0.88)	0.53 (0.42 to 0.69)
Coefficient (95% CI) of externalizing behavior score, age 3-5 y^b^	0.2 (−0.1 to 0.5)	[Reference]	0.5 (0.3 to 0.8)	1.3 (1.0 to 1.6)	2.1 (1.7 to 2.5)

Abbreviation: OR, odds ratio.

^a^ Data from the 2018-2021 National Survey of Children’s Health.

^b^ Adjusted for age, sex, race and ethnicity, caregiver’s education, caregiver’s marital status, caregiver’s mental and emotional health, caregiver having emotional support for parenting, federal poverty level, home language, and neighborhood support. Complete models are presented in eTable 3 in Supplement 1.

The 4-year prevalence of high screen time was 73.8% (95% CI, 70.6%-76.8%) among children with ASD and 58.6% (95% CI, 56.7%-60.6%) among children with developmental delays (but without ASD). The inverse association trend between screen time and flourishing and the positive association trend between screen time and the externalizing behavior score were observed in these subgroups (eTable 4 and eTable 5 in Supplement 1).

## Discussion

We found that, overall, the prevalence of high screen time increased in the first pandemic year (2020) as compared to the prepandemic years (2018 and 2019), but it returned to prepandemic levels in 2021 in a nationally representative sample of young US children. Among children living in families with federal poverty level below 100%, however, screen time did not return to prepandemic levels in 2021. Children aged 3 to 5 years who had 2 hours per day or more of screen time were less likely to flourish and had higher externalizing behavior scores, compared to those who had 1 hour per day of screen time. We found no association between screen time and flourishing among children aged 6 months to 2 years.

How the COVID-19 pandemic has affected young children’s screen time is an ongoing public health interest. Hedderson et al^4^ reported that screen time remained 1.1 hours per day higher in the first half of 2021 as compared to the prepandemic period among US children aged 4 to 12 years, primarily because increased screen time for recreation and game play did not return to prepandemic levels. To our knowledge, the present study is one of the first to provide population-based evidence that screen time overall returned to prepandemic levels in 2021 after a rise in 2020 among US children aged 2 to 5 years. Despite this overall trend, it is important to highlight that for children living in families with federal poverty level below 100%, who were already at high risk of high screen time in the prepandemic period,^34^ the high screen time prevalence did not return to prepandemic levels in 2021. We also found that, although the prevalence of high screen time did not increase from 2019 to 2020 among non-Hispanic Black children, there was an increase from 2020 to 2021, revealing that nearly 3 in 4 young non-Hispanic Black children had high screen time in 2021. High screen time observed among non-Hispanic Black children is consistent with prior studies.^4,35^ Continuous surveillance of screen time in 2022 and beyond is required to address health disparities and plan targeted interventions. Screen time interventions among young children must also consider sociodemographic risk factors for excessive screen use.^35^

We found no significant changes in the prevalence of high screen time during 2018 to 2021 among US children aged 6 months to 1 year. However, we found that the prevalence of high screen time was approximately 30% higher among 1-year-olds, compared to children 6 to 12 months of age. Recent longitudinal studies^36,37^ suggest that screen exposure substantially increases at age 1 year, by a half an hour from age 12 to 18 months and another half hour to 24 months. It appears that 1 year of age is a critical period to target for healthy screen use. As suggested by the AAP guidelines,^30^ it is important to introduce high-quality programing and applications with close parental monitoring (eg, use media together) at this age.

High screen time is postulated to influence child development and well-being, partly by replacing learning opportunities that foster well-being.^15^ Fast-paced and intense audiovisual screen media may also impede self-regulation that leads to impulsivity and attention problems.^27,38,39,40,41,42^ We found no association between screen time and flourishing among children aged 6 months to 2 years; however, children aged 3 to 5 years who had 2 or more hours per day of screen time were less likely to flourish and had higher externalizing behavior scores, compared to those who had 1 hour per day of screen time, consistent with prior study findings.^17,29^ Of the 4 flourishing dimensions, resilience and curiosity, both of which are related to psychological adjustment,^43,44^ drove theflourishing-screen time association. The consistent findings among children aged 3 to 5 years with ASD and developmental delay assure that the inverse association between screen time and psychological well-being are relevant to broader preschool-aged populations. However, our cross-sectional examination does not rule out a possible reverse causation (eg, children with externalizing behaviors engage in more screen use^45^). It is interesting that children aged 3 to 5 years with less than 1 hour per day of screen time were less likely to flourish, compared to their counterparts with 1 hour per day of screen time, which suggests that some screen exposure (ie, 1 h/d) could be positive for preschool-aged children’s psychological well-being. Prospective studies should investigate this potentially nonlinear dose-response relationship with a continuous screen time measurement to establish evidence-based screen use guidelines.

### Strengths and Limitations

This is one of the largest screen time investigations that includes data from the 2-year pandemic period from a nationally representative sample of young US children. Limitations are related to screen time measurement. As the NSCH measured screen time incrementally, instead of continuously, the screen time categorization may not be accurately aligned with the AAP screen use recommendations.^30^ A single screen time question for all types of screen use did not allow us to account for the type of screen content, which could have confounded the associations with psychological well-being.^39,40,42^ Additionally, proxy-reported measures for screen time and psychological well-being could have caused measurement error.^46^ This nondifferential misclassification was likely to bias estimates toward the null. Future research using objective measures of screen use should help strengthen the evidence for these associations.^47^ This study was unable to consider online schooling during the pandemic in screen use examination because online schooling data were unavailable. The cross-sectional investigation limits our ability to establish a temporal relationship. The results derived after excluding missing data may have underestimated the magnitude of racial and ethnic and income disparities. Interpreting clinical significance of the externalizing behavior score is limited because there was no established clinical significance or interpretation for the externalizing behavior score used.

## Conclusions

In this multiyear cross-sectional study of a representative sample of young children in the US, the increased prevalence of high screen time in 2020 returned to prepandemic levels in 2021 among young American children; however, its prevalence remained elevated in a subgroup of children living in families with federal poverty level below 100%. Two hours or more of daily screen time was associated with lower psychological well-being among preschool-aged children. These findings highlight urgent needs to provide support for healthy screen use to families with young children.

## Notes

Supplement 1. **eTable 1.** Comparison of Demographic Characteristics Between Participants Aged 6 Months to 5 Years Who Were Included in Analyses and Participants Who Were Excluded Due to Missing Data. The 2018 to 2021 National Survey of Children’s Health **eTable 2.** Trends of Flourishing and Externalizing Behavior Scores Over Survey Years Among Participants Aged 6 Months to 5 Years. The 2018 to 2021 National Survey of Children’s Health **eTable 3.** Multivariable Regression Models to Predict Flourishing and Externalizing Behavior Score Among Participants Aged 6 Months to 5 Years. The 2018 to 2021 National Survey of Children’s Health **eTable 4.** Multivariable Regression Models to Predict Flourishing and Externalizing Behavior Score Among Participants Aged 3 to 5 Years With Autism Spectrum Disorder. The 2018 to 2021 National Survey of Children’s Health **eTable 5.** Multivariable Regression Models to Predict Flourishing and Externalizing Behavior Score Among Participants Aged 6 Months to 5 Years With Developmental Delays but Without Autism Spectrum Disorder. The 2018 to 2021 National Survey of Children’s Health

Supplement 2.
Data Sharing Statement
