# Supplementary material for: Screen Time, Sociodemographic Factors, and Psychological Well-Being Among Young Children
Source: JAMA Netw Open. 2024 Mar 5;7(3):e2354488. doi: 10.1001/jamanetworkopen.2023.54488 (PMC10915694; doi:10.1001/jamanetworkopen.2023.54488)
Supplement: Supplement 1. — eTable 1. Comparison of Demographic Characteristics Between Participants Aged 6 Months to 5 Years Who Were Included in Analyses and Participants Who Were Excluded Due to Missing Data. The 2018 to 2021 National Survey of Children’s Health eTable 2. Trends of Flourishing and Externalizing Behavior Scores Over Survey Years Among Participants Aged 6 Months to 5 Years. The 2018 to 2021 National Survey of Children’s Health eTable 3. Multivariable Regression Models to Predict Flourishing and Externalizing Behavior Score Among Participants Aged 6 Months to 5 Years. The 2018 to 2021 National Survey of Children’s Health eTable 4. Multivariable Regression Models to Predict Flourishing and Externalizing Behavior Score Among Participants Aged 3 to 5 Years With Autism Spectrum Disorder. The 2018 to 2021 National Survey of Children’s Health eTable 5. Multivariable Regression Models to Predict Flourishing and Externalizing Behavior Score Among Participants Aged 6 Months to 5 Years With Developmental Delays but Without Autism Spectrum Disorder. The 2018 to 2021 National Survey of Children’s Health [file jamanetwopen-e2354488-s001.pdf]

Supplemental Online Content

Kwon S, Armstrong B, Wetoska N, Capan S. Sociodemographic factors, screen time, and reported behaviors among young children. *JAMA Netw Open*. 2024;7(2):e2354488. doi:10.1001/jamanetworkopen.2023.54488

eTable 1. Comparison of Demographic Characteristics Between Participants Aged 6 Months to 5 Years Who Were Included in Analyses and Participants Who Were Excluded Due to Missing Data. The 2018 to 2021 National Survey of Children's Health
eTable 2. Trends of Flourishing and Externalizing Behavior Scores Over Survey Years Among Participants Aged 6 Months to 5 Years. The 2018 to 2021 National Survey of Children's Health
eTable 3. Multivariable Regression Models to Predict Flourishing and Externalizing Behavior Score Among Participants Aged 6 Months to 5 Years. The 2018 to 2021 National Survey of Children's Health
eTable 4. Multivariable Regression Models to Predict Flourishing and Externalizing Behavior Score Among Participants Aged 3 to 5 Years With Autism Spectrum Disorder. The 2018 to 2021 National Survey of Children's Health
eTable 5. Multivariable Regression Models to Predict Flourishing and Externalizing Behavior Score Among Participants Aged 6 Months to 5 Years With Developmental Delays but Without Autism Spectrum Disorder. The 2018 to 2021 National Survey of Children's Health

This supplemental material has been provided by the authors to give readers additional information about their work.

eTable 1. Comparison of Demographic Characteristics Between Participants Aged 6 Months to 5 Years Who Were Included in Analyses and Participants Who Were Excluded Due to Missing Data. The 2018 to 2021 National Survey of Children's Health

	Included (n=48775)	Excluded (n=835)
Age, mean (95% CI)	2.6 (2.5, 2.6)	2.2 (1.9, 2.5)
Female sex, % (95% CI)	48.9 (47.9, 49.9)	46.9 (39.1, 54.7)
Race/ethnicity, % (95% CI)		
Hispanic	24.5 (23.4, 25.5)	34.6 (25.5, 43.7)
Non-Hispanic Asian	4.7 (4.3, 5.0)	2.8 (1.7, 3.8)
Non-Hispanic Black	12.1 (11.4, 12.9)	18.8 (13.7, 23.8)
Non-Hispanic White	51.4 (50.4, 52.4)	37.7 (30.7, 44.6)
Federal poverty level, % (95% CI)		
<100% (below poverty)	16.6 (15.7, 17.5)	24.1 (15.7, 32.6)
100 to <200%	22.0 (21.1, 22.9)	36.9 (29.1, 44.6)
200 to <400%	32.4 (31.5, 33.3)	35.3 (28.6, 42.0)
≥400%	29.0 (28.2, 29.8)	3.7 (2.1, 5.3)

CI, confidence interval. The National Survey of Children's Health was funded and directed by US Maternal and Child Health Bureau of the Health Resources and Services Administration and fielded by the US Census Bureau.

© 2024 Kwon S et al. *JAMA Network Open.*

eTable 2. Trends of Flourishing and Externalizing Behavior Scores Over Survey Years Among Participants Aged 6 Months to 5 Years. The 2018 to 2021 National Survey of Children's Health

	2018	2019	2020	2021
Flourishing, % (95% CI)	84.6 (82.6, 86.4)	83.9 (81.9, 85.8)	82.9 (81.4, 84.5)	82.3 (80.9, 83.7)
Externalizing behavior score, mean (95% CI)[a]	13.4 (13.2, 13.6)	13.6 (13.3, 13.8)	14.1 (13.9, 14.3)	13.9 (13.8, 14.1)

CI, confidence interval. The National Survey of Children's Health was funded and directed by US Maternal and Child Health Bureau of the Health Resources and Services Administration and fielded by the US Census Bureau.

[a]Externalizing behavior score was assessed among participants aged 3-5 years.

© 2024 Kwon S et al. *JAMA Network Open.*

eTable 3. Multivariable Regression Models to Predict Flourishing and Externalizing Behavior Score Among Participants Aged 6 Months to 5 Years. The 2018 to 2021 National Survey of Children's Health

	Flourishing (age 6 months to 2 years; n=26496)	Flourishing (age 3-5 years; n=26400)	Externalizing behavior score (age 3-5 years; n=25747)
	OR (95% CI)	OR (95% CI)	Coefficient (95% CI)
Intercept	NA	NA	14.3 (13.7, 14.8)
Age in years	1.15 (0.98, 1.35)	1.01 (0.91, 1.11)	-0.5 (-0.7, -0.4)
Sex			
Male vs. female	1.02 (0.84, 1.24)	0.81 (0.69, 0.95)	0.7 (0.5, 0.9)
Race/ethnicity			
Hispanic vs. Non-Hispanic White	0.74 (0.55, 0.99)	0.95 (0.74, 1.23)	-0.5 (-0.8, -0.1)
Non-Hispanic Asian vs. Non-Hispanic White	0.68 (0.44, 1.04)	0.60 (0.44, 0.80)	0.2 (-0.2, 0.6)
Non-Hispanic Black vs. Non-Hispanic White	0.64 (0.46, 0.89)	1.09 (0.84, 1.41)	-0.3 (-0.7, 0.05)
Non-Hispanic multi-race vs. Non-Hispanic White	0.88 (0.64, 1.22)	1.21 (0.94, 1.55)	-0.1 (-0.4, 0.2)
Other vs. Non-Hispanic White	0.72 (0.34, 1.53)	1.05 (0.59, 1.86)	-0.3 (-1.2, 0.6)
Caregiver's education			
≤High school vs. 4-year college degree	0.63 (0.48, 0.83)	0.71 (0.57, 0.88)	0.1 (-0.2, 0.4)
Technical school or some college vs. 4-year college degree	1.01 (0.79, 1.30)	0.79 (0.66, 0.96)	-0.03 (-0.3, 0.2)
Caregiver's marital status			
Not married, but living with a partner vs. married	0.88 (0.62, 1.25)	1.16 (0.81, 1.66)	0.4 (-0.03, 0.9)
Never married vs. married	1.07 (0.74, 1.54)	0.91 (0.68, 1.22)	0.9 (0.4, 1.4)
Divorced/separated/widowed vs. married	1.14 (0.73, 1.78)	1.24 (0.95, 1.62)	0.3 (-0.1, 0.7)
Missing vs. married	1.08 (0.25, 4.68)	0.63 (0.22, 1.82)	-0.4 (-1.1, 0.4)
Caregiver's mental and emotional health			
Poor to fair vs. excellent to good	0.48 (0.32, 0.70)	0.69 (0.54, 0.90)	1.6 (1.2, 2.0)
Missing vs. excellent to good	1.45 (0.32, 6.51)	1.03 (0.32, 3.32)	1.1 (-0.1, 2.2)
Caregiver having emotional support for parenting			
No vs. yes	0.59 (0.45, 0.77)	0.72 (0.59, 0.89)	0.1 (-0.3, 0.4)
Missing vs. yes	0.66 (0.27, 1.63)	1.48 (0.55, 3.95)	0.8 (-0.3, 2.0)
Federal poverty level			
<100 (below poverty) vs. ≥400%	0.80 (0.59, 1.10)	0.63 (0.47, 0.83)	0.5 (0.01, 0.9)
100- <200 vs. ≥400%	0.88 (0.66, 1.17)	0.94 (0.74, 1.19)	-0.1 (-0.4, 0.2)
200- <400 vs. ≥400%	1.10 (0.86, 1.42)	1.10 (0.92, 1.32)	0.1 (-0.1, 0.3)
Home language			
Non-English vs. English	0.54 (0.38, 0.75)	0.54 (0.41, 0.72)	0.1 (-0.3, 0.6)
Supportive neighborhood			
No vs. yes	0.76 (0.62, 0.94)	0.80 (0.67, 0.94)	0.8 (0.6, 1.1)
Missing vs. yes	0.47 (0.20, 1.11)	0.67 (0.34, 1.32)	0.6 (-0.6, 1.7)
Screen Time			

© 2024 Kwon S et al. *JAMA Network Open.*

<1 vs. 1 hour/day	1.26 (0.95, 1.66)	0.66 (0.52, 0.85)	0.2 (-0.1, 0.5)
2 vs. 1 hour/day	1.15 (0.83, 1.59)	0.81 (0.66, 0.99)	0.5 (0.3, 0.8)
3 vs. 1 hour/day	0.76 (0.53, 1.09)	0.68 (0.52, 0.88)	1.3 (1.0, 1.6)
≥4 vs. 1 hour/day	1.08 (0.74, 1.58)	0.53 (0.42, 0.69)	2.1 (1.7, 2.5)

CI, confidence interval; NA, not applicable; OR, odds ratio; the "other" race/ethnicity category includes American Indian or Alaska Native, Native Hawaiian and other Pacific Islander. The National Survey of Children's Health was funded and directed by US Maternal and Child Health Bureau of the Health Resources and Services Administration and fielded by the US Census Bureau.

© 2024 Kwon S et al. *JAMA Network Open.*

eTable 4. Multivariable Regression Models to Predict Flourishing and Externalizing Behavior Score Among Participants Aged 3 to 5 Years With Autism Spectrum Disorder. The 2018 to 2021 National Survey of Children's Health

	Flourishing (n=530)	Externalizing behavior score (n=521)*
	OR (95% CI)	Coefficient (95% CI)
Intercept	NA	17.4 (15.3, 19.5)
Age in years	1.04 (0.82, 1.32)	-0.1 (-0.5, 0.3)
Sex		
Male vs. female	1.05 (0.67, 1.66)	0.1 (-0.7, 1.0)
Race/ethnicity		
Hispanic vs. Non-Hispanic White	1.99 (1.14, 3.46)	-3.4 (-7.3, 0.5)
Non-Hispanic Asian vs. Non-Hispanic White	2.17 (0.96, 4.91)	-2.0 (-3.3, -0.8)
Non-Hispanic Black vs. Non-Hispanic White	1.44 (0.71, 2.93)	-2.0 (-3.2, -0.8)
Non-Hispanic multi-race vs. Non-Hispanic White	1.90 (0.99, 3.65)	-1.1 (-2.3, 0.1)
Other vs. Non-Hispanic White	0.99 (0.08, 12.02)	-0.5 (-1.5, 0.5)
Caregiver's education		
≤High school vs. 4-year college degree	0.85 (0.44, 1.63)	1.6 (0.5, 2.8)
Technical school or some college vs. 4-year college degree	1.14 (0.72, 1.81)	0.7 (-0.1, 1.5)
Caregiver's marital status		
Not married, but living with a partner vs. married	1.30 (0.70, 2.40)	0.5 (-0., 1.6)
Never married vs. married	1.35 (0.68, 2.68)	0.5 (-0.7, 1.7)
Divorced/separated/widowed vs. married	0.49 (0.24, 1.01)	0.6 (-0.5, 1.8)
Missing vs. married	0.54 (0.11, 2.72)	1.6 (-1.2, 4.3)
Caregiver's mental and emotional health		
Poor to fair vs. excellent to good	0.55 (0.30, 1.00)	1.8 (0.8, 2.7)
Caregiver having emotional support for parenting:		
No vs. yes	0.90 (0.56, 1.45)	0.7 (-0.1, 1.5)
Federal poverty level		
<100 (below poverty) vs. ≥400%	0.58 (0.28, 1.21)	1.4 (0.1, 2.6)
100- <200 vs. ≥400%	0.81 (0.49, 1.36)	-0.04 (-1.0, 0.9)
200- <400 vs. ≥400%	0.93 (0.57, 1.53)	0.4 (-0.5, 1.2)
Home language		
Non-English vs. English	0.49 (0.23, 1.03)	-0.6 (-1.9, 0.7)
Supportive neighborhood		
No vs. yes	0.55 (0.37, 0.81)	1.3 (0.6, 2.0)
Missing vs. yes	0.77 (0.21, 2.79)	-1.0 (-3.3, 1.3)
Screen Time		
<1 vs. 1 hour/day	1.13 (0.54, 2.37)	0.3 (-1.0, 1.7)
2 vs. 1 hour/day	0.76 (0.44, 1.32)	0.7 (-0.3, 1.7)
3 vs. 1 hour/day	0.71 (0.38, 1.33)	0.7 (-0.4, 1.8)
≥4 vs. 1 hour/day	0.62 (0.33, 1.18)	1.0 (-0.1, 2.1)

CI, confidence interval; NA, not applicable; OR, odds ratio; the "other" race/ethnicity category includes American Indian or Alaska Native, Native Hawaiian and other Pacific Islander; multivariable linear

© 2024 Kwon S et al. *JAMA Network Open.*

regression analysis for age 6 months to 2 years was not conducted due to a small sample size (n=83). The National Survey of Children's Health was funded and directed by US Maternal and Child Health Bureau of the Health Resources and Services Administration and fielded by the US Census Bureau.

© 2024 Kwon S et al. *JAMA Network Open.*

eTable 5. Multivariable Regression Models to Predict Flourishing and Externalizing Behavior Score Among Participants Aged 6 Months to 5 Years With Developmental Delays but Without Autism Spectrum Disorder. The 2018 to 2021 National Survey of Children's Health

	Flourishing (age 6 months to 2 years; n=659)	Flourishing (age 3-5 years; n=1220)	Externalizing behavior score (n=1199)
	OR (95% CI)	OR (95% CI)	Coefficient (95% CI)
Intercept	NA	NA	13.7 (12.4, 15.1)
Age in years	1.00 (0.74, 1.34)	0.83 (0.72, 0.97)	0.1 (-0.2, 0.4)
Sex			
Male vs. female	1.23 (0.85, 1.78)	0.86 (0.66, 1.12)	0.7 (0.2, 1.2)
Race/ethnicity			
Hispanic vs. Non-Hispanic White	1.16 (0.64, 2.07)	0.84 (0.57, 1.24)	0.03 (-1.8, 1.9)
Non-Hispanic Asian vs. Non-Hispanic White	1.15 (0.48, 2.75)	1.46 (0.74, 2.91)	0.5 (-0.8, 1.7)
Non-Hispanic Black vs. Non-Hispanic White	0.50 (0.24, 1.05)	1.29 (0.77, 2.16)	0.2 (-0.8, 1.1)
Non-Hispanic multi-race vs. Non-Hispanic White	0.95 (0.49, 1.85)	0.60 (0.38, 0.94)	-0.3 (-1.2, 0.5)
Other vs. Non-Hispanic White	0.90 (0.19, 4.28)	2.49 (0.69, 8.98)	-0.4 (-1.1, 0.4)
Caregiver's education			
≤high school vs. 4-year college degree	0.39 (0.22, 0.69)	1.04 (0.67, 1.60)	1.0 (0.2, 1.8)
Technical school or some college vs. 4-year college degree	1.24 (0.78, 1.97)	1.01 (0.75, 1.37)	0.4 (-0.1, 1.0)
Caregiver's marital status			
Not married, but living with a partner vs. married	1.12 (0.58, 2.18)	1.17 (0.72, 1.92)	0.7 (-0.2, 1.7)
Never married vs. married	1.15 (0.55, 2.41)	0.76 (0.46, 1.24)	0.3 (-0.6, 1.3)
Divorced/separated/widowed vs. married	1.09 (0.51, 2.35)	0.84 (0.54, 1.30)	0.2 (-0.7, 1.0)
Missing vs. married	1.61 (0.25, 10.5)	0.84 (0.29, 2.45)	0.4 (-1.6, 2.5)
Caregiver's mental and emotional health			
Poor to fair vs. excellent to good	0.60 (0.35, 1.03)	0.84 (0.58, 1.22)	1.4 (0.7, 2.1)
Caregiver having emotional support for parenting:			
No vs. yes	0.92 (0.55, 1.54)	0.66 (0.48, 0.92)	0.9 (0.3, 1.6)
Federal poverty level			
<100 (below poverty) vs. ≥400%	0.76 (0.41, 1.39)	0.56 (0.36, 0.86)	1.3 (0.5, 2.2)
100- <200 vs. ≥400%	0.75 (0.41, 1.39)	0.91 (0.64, 1.31)	0.2 (-0.5, 0.9)
200- <400 vs. ≥400%	1.16 (0.73, 1.83)	0.85 (0.63, 1.16)	0.4 (-0.1, 1.0)
Home language			
Non-English vs. English	1.26 (0.51, 3.13)	0.66 (0.36, 1.21)	-0.3 (-1.5, 0.9)
Supportive neighborhood			
No vs. yes	0.71 (0.49, 1.04)	0.70 (0.54, 0.90)	1.0 (0.5, 1.5)
Missing vs. yes	0.83 (0.12, 5.61)	0.94 (0.31, 2.87)	1.7 (-0.3, 3.8)
Screen Time			
<1 vs. 1 hour/day	0.86 (0.50, 1.45)	1.06 (0.68, 1.66)	-0.2 (-1.0, 0.6)
2 vs. 1 hour/day	1.08 (0.62, 1.88)	0.94 (0.67, 1.31)	0.8 (0.2, 1.4)
3 vs. 1 hour/day	0.61 (0.32, 1.15)	0.77 (0.52, 1.14)	1.1 (0.3, 1.8)

© 2024 Kwon S et al. *JAMA Network Open.*

≥4 vs. 1 hour/day	0.73 (0.38, 1.41)	0.63 (0.42, 0.96)	1.9 (1.1, 2.7)

CI, confidence interval; NA, not applicable; OR, odds ratio; the “other” race/ethnicity category includes American Indian or Alaska Native, Native Hawaiian and other Pacific Islander. The National Survey of Children’s Health was funded and directed by US Maternal and Child Health Bureau of the Health Resources and Services Administration and fielded by the US Census Bureau.

© 2024 Kwon S et al. *JAMA Network Open.*
