# Supplementary material for: Screen Time, Sociodemographic Factors, and Psychological Well-Being Among Young Children
Source: JAMA Netw Open. 2024 Mar 5;7(3):e2354488. doi: 10.1001/jamanetworkopen.2023.54488 (PMC10915694; doi:10.1001/jamanetworkopen.2023.54488)
Supplement: Supplement 2. — Data Sharing Statement [file jamanetwopen-e2354488-s002.pdf]

Data Sharing Statement

Kwon. Screen Time, Sociodemographic Factors, and Psychological Well-Being Among Young Children. *JAMA Netw Open*. Published March 05, 2024.
doi:10.1001/jamanetworkopen.2023.54488

Data
Data available: No

Additional Information
Explanation for why data not available: The data are publicly available.
